# The expression of 11β-hydroxysteroid dehydrogenase type 1 is increased in experimental periodontitis in rats

**DOI:** 10.1186/s12903-016-0303-z

**Published:** 2016-10-03

**Authors:** Takaya Nakata, Makoto Umeda, Hiroaki Masuzaki, Hirofumi Sawai

**Affiliations:** 1 Department of Periodontology, Graduate School of Dentistry, Osaka Dental University, Hirakata, Osaka Japan; 2 Department of Periodontology, Osaka Dental University, Hirakata, Osaka Japan; 3 Division of Endocrinology, Diabetes and Metabolism, Hematology, Rheumatology, Graduate School of Internal Medicine, University of the Ryukyus, Nakagami-gun, Okinawa, Japan; 4 Department of Internal Medicine, Osaka Dental University, Hirakata, Osaka Japan

**Keywords:** 11β-Hydroxysteroid dehydrogenase type 1, Glucocorticoids, Chronic periodontitis, Experimental periodontitis, Inflammation

## Abstract

**Background:**

The involvement of 11β-hydroxysteroid dehydrogenase type 1 (11β-HSD1), which converts inactive glucocorticoids into active glucocorticoids intracellularly, in metabolic diseases and chronic inflammatory diseases has been elucidated. We recently reported that an increase in 11β-HSD1 expression was associated with chronic periodontitis in humans irrespective of obesity. To further clarify the role of 11β-HSD1 in chronic periodontitis, the expression of 11β-HSD1 was investigated in experimental periodontitis model in rats.

**Methods:**

Experimental periodontitis was induced by silk ligature of left maxillary second molars of 7-week-old male Wistar rats, and periodontal tissues were collected at day 3. The expression of 11β-HSD1, 11β-HSD2, and TNFα mRNA was examined using real time reverse transcription-polymerase chain reaction. The expression of TNFα was used as an indicator of inflammation. Thus, the rats in which the levels of TNFα mRNA were increased in the ligature-induced periodontitis compared with the control were analysed.

**Results:**

The findings demonstrated that the expression of 11β-HSD1 mRNA was significantly increased in experimental periodontitis compared with the control. The increase in the levels of 11β-HSD1 mRNA in the ligature-induced periodontitis compared with the control was positively correlated with that of TNFα mRNA. On the other hand, the expression of 11β-HSD2 mRNA, which inactivates glucocorticoids, was slightly decreased in experimental periodontitis. Therefore, the ratio of 11β-HSD1 versus 11β-HSD2 mRNA was significantly higher in experimental periodontitis than in the control.

**Conclusions:**

These results suggest that the increased expression of 11β-HSD1, which would result in the increased levels of intracellular glucocorticoids, may play a role in the pathophysiology of experimental periodontitis.

## Background

The involvement of cortisol, a major glucocorticoid hormone in humans, in chronic periodontitis has been investigated. Serum and salivary cortisol levels were reported to be correlated with measures of chronic periodontitis [1–5]. In animal experiments, ligature-induced periodontitis was more severe in hypothalamic-pituitary-adrenal (HPA) high-responding Fischer 344 rats than in MHC-identical but HPA low-responding Lewis rats. Treatment with RU 486, a glucocorticoid receptor antagonist, reduced experimental periodontitis in Fischer 344 rats, suggesting that the increased glucocorticoids play a role in experimental periodontitis [6–8]. However, the mechanism by which the levels of glucocorticoids are increased and the role of the increased glucocorticoids in chronic periodontitis remain to be determined.

11β-hydroxysteroid dehydrogenases, one of the many enzymes involved in the metabolism of glucocorticoids, catalyze the intracellular conversion between active (cortisol in humans and corticosterone in rodents) and inactive (cortisone in humans and 11-dehydrocorticosterone in rodents) glucocorticoids [9–12]. Generally, 11β-HSD1 activates and 11β-hydroxysteroid dehydrogenase type 2 (11β-HSD2) inactivates glucocorticoids. It was reported that the expression of 11β-HSD1 was increased in adipocytes, especially in visceral fat, of patients with metabolic diseases, which suggests that 11β-HSD1 plays a role in the pathogenesis of metabolic diseases [13–15]. Animal studies supported this notion. 11β-HSD1-deficient mice showed attenuated glucocorticoid-inducible responses and resisted hyperglycemia on obesity or stress [16], whereas transgenic mice overexpressing 11β-HSD1 selectively in adipose tissue developed visceral obesity that was exaggerated by a high-fat diet [17, 18]. Furthermore, several 11β-HSD1 inhibitors were reported to ameliorate metabolic diseases in mice [19, 20]. Currently, several 11β-HSD1 inhibitors are being developed for the treatment of metabolic diseases, especially type 2 diabetes mellitus [21–24].

Since obesity, as well as metabolic diseases, can be regarded as chronic inflammation of adipose tissue [25, 26], the involvement of 11β-HSD1 in other chronic inflammatory diseases has been investigated. The increased expression of 11β-HSD1 was demonstrated in inflammatory bowel diseases and rheumatoid arthritis [27–30], suggesting that the increased 11β-HSD1 plays a role in chronic inflammation. We recently investigated the expression of 11β-HSD1 in periodontal tissues from patients with chronic periodontitis, and reported that the expression of 11β-HSD1, as well as the ratio of 11β-HSD1 versus 11β-HSD2, was significantly higher in chronic periodontitis than in the control irrespective of obesity [31]. In this study, to further clarify the role of 11β-HSD1 in chronic periodontitis, the expression of 11β-HSD1 was investigated in ligature-induced experimental periodontitis in rats.

## Methods

### Animals

Fifty six-week-old male Wistar rats were purchased from Shimizu Laboratory Supplies (Kyoto, Japan) and were maintained under specific pathogen-free conditions with food and distilled water at Osaka Dental University Animal Care. The experimental protocol was approved by the Committee for Animal Experiments of Osaka Dental University (#14-09001), and experimental procedures were performed in accordance with the Guidelines for Animal Experiments of Osaka Dental University.

### Experimental periodontitis

Experimental periodontitis was induced as described with modifications [32]. Experiments were started at 7 weeks of age. General anesthesia was induced with inhalation of isoflurane (Wako Pure Chemical Industries, Osaka, Japan) and intraperitoneal injections of pentobarbital (Kyoritsu Seiyaku Corporation, Tokyo, Japan) at 0.3 mg/kg body weight. Silk threads (5-0 Nescosuture, Alfresa Pharma Corporation, Osaka, Japan) were ligated around the cervix of the left maxillary second molars to induce experimental periodontitis. The right maxillary second molars were used as the control. Three days after the ligation, rats were euthanized with an overdose of isoflurane and pentobarbital.

### Micro-CT analysis

Images of the excised maxillary bones were analysed using a micro-CT scanner, SMX-130CT (Shimadzu Corporation, Kyoto, Japan). The micro-CT parameters were set as follows: image pixel size, 512 × 512; voltage, 40 kV; beam current, 40 μA; view number, 1200; scaling coefficient, 10. Three dimensional images were generated using VGStudio MAX 1.2.1 software (Volume Graphics, GmbH, Germany).

### RNA extraction

Periodontal tissues around the right and left maxillary second molars (for the control and periodontitis group, respectively) were excised, and then homogenized in 1 ml of TRIzol Reagent (Invitrogen, Carlsbad, CA, USA). After the addition of 0.2 ml chloroform, the mixture was centrifuged at 12,000 *g* for 15 min at 4 °C. The aqueous phase was transferred to a new tube and 0.5 ml of isopropyl alcohol was added. After centrifugation at 10,000 *g* for 10 min at 4 °C, the RNA precipitate was washed with 1 ml of 75 % ethanol and centrifuged at 7500 *g* for 5 min at 4 °C. Then the RNA precipitate was air-dried and dissolved in RNase-free water. For determination of RNA concentration, the absorbance at 260 nm was measured using a spectrophotometer, SmartSpec 3000 (Bio-Rad, Hercules, CA, USA).

### Real time reverse transcription (RT)-polymerase chain reaction (PCR)

As probes for PCR, TaqMan Gene Expression Assays for rat 11β-HSD1 (#Rn00567167_m1), rat 11β-HSD2 (#Rn00492539_m1), rat TNFα (#Rn99999017_m1), and rat glyceraldehyde-3-phosphate dehydrogenase (GAPDH) (#Rn01775763_g1) were purchased from Applied Biosystems (Foster City, CA, USA). RT-PCR was performed using TaqMan RNA-to-C_T_ 1-Step Kit and StepOnePlus Real Time PCR System (Applied Biosystems, Foster City, CA, USA). Each template (20 ng RNA) was mixed with 1 μl of probe, 0.5 μl of RT Enzyme Mix, 10 μl of RT-PCR Mix, and Nuclease-free water was added so that the total volume of the mixture should be 20 μl. The mixture was incubated for 15 min at 48 °C for reverse transcription, followed by incubation for 10 min at 95 °C for inactivation of reverse transcriptase and activation of DNA polymerase. For PCR, the mixture was incubated for 15 s at 95 °C for denaturing and then for 1 min at 60 °C for annealing/extension for 50 cycles. The values of 11β-HSD1, 11β-HSD2, and TNFα mRNA relative to GAPDH mRNA were calculated in each sample.

### Histological examinations

Another set of experiments was performed for histological examinations. After euthanasia, the rats were perfused with 10 % formaldehyde neutral buffer solution (Sigma-Aldrich, St. Louis, MO, USA). Then the maxillary bones were excised, and fixed in 10 % formaldehyde neutral buffer solution at 4 °C for 3 days. The bones were decalcified in a rapid decalcification solution, K-CX (Falma, Tokyo, Japan), at 4 °C for 24 h, followed by conventional dehydration and paraffin embedding. After cutting into 5 μm-thick sections, the specimens were deparaffinized and then stained with hematoxylin-eosin (HE) or immunostained with either anti-11β-HSD1 antibody (Bioss, Woburn, MA, USA) or anti-11β-HSD2 antibody (Santa Cruz Biotechnology, Dallas, TX, USA) using Envision + kit/HRP (DAB) (Dako, Glostrup, Denmark). Images were obtained using an all-in-one microscope, BZ-9000 (Keyence, Osaka, Japan).

### Statistical analyses

The comparison between the control and the periodontitis group was assessed by paired *t*-test. The correlation between the increase in the levels of 11β-HSD1 mRNA and that of TNFα mRNA was assessed by Pearson’s correlation analysis using SPSS software version 21.0 (IBM, Armonk, NY, USA).

## Results

**Fig. 1:**
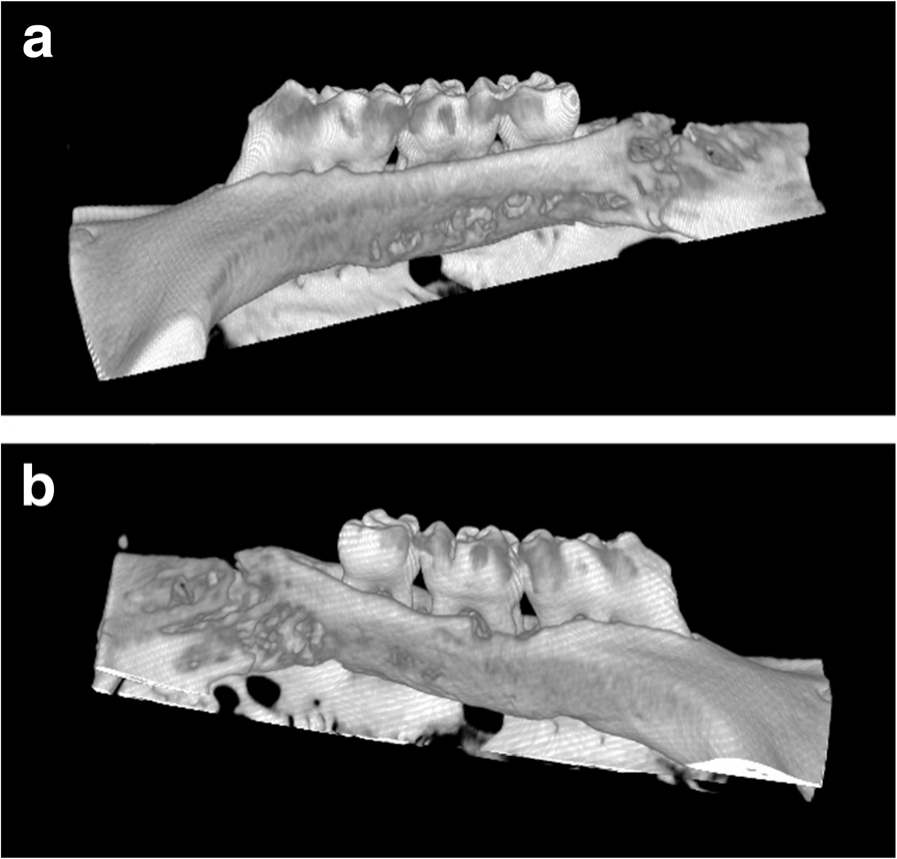
Micro-CT scan of maxillary bones on the palatal side. **a** Control. **b** Ligature-induced periodontitis (after 2 weeks)

Experimental periodontitis was induced by ligature placement, and alveolar bone loss due to ligature-induced periodontitis was observed after 2 weeks (Fig. 1).

**Fig. 2:**
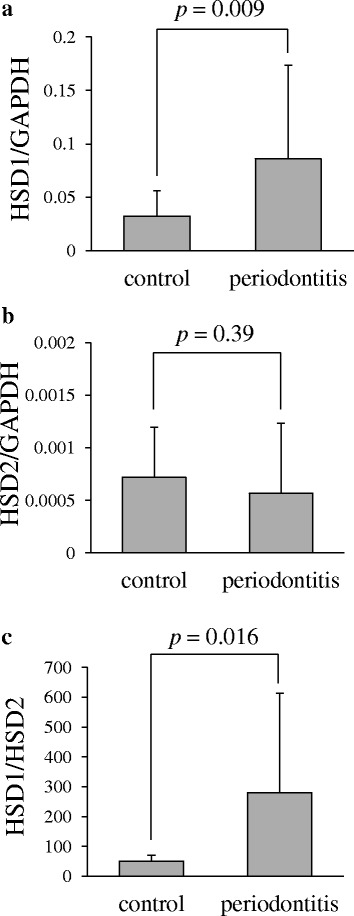
The expression of 11β-HSD1 mRNA is increased in the ligature-induced periodontitis compared with the control. **a** The values of 11β-HSD1 mRNA relative to GAPDH mRNA. **b** The values of 11β-HSD2 mRNA relative to GAPDH mRNA. **c** The ratio of 11β-HSD1 versus 11β-HSD2 mRNA. Values are expressed as mean + standard deviation (*n* = 16)

To investigate the involvement of 11β-HSD1 in experimental periodontitis, the expression of 11β-HSD1, as well as 11β-HSD2 mRNA, in periodontal tissues was analysed by the real time RT-PCR method. The expression of TNFα mRNA in periodontal tissues was concurrently examined to confirm the induction of periodontitis by ligature placement. Thus, the data of rats (*n* = 16) in which the levels of TNFα mRNA were increased more than 1.4-fold in the ligature-induced periodontitis compared with the control were analysed. The levels of 11β-HSD1 mRNA were significantly higher in periodontal tissues around the left maxillary second molars than in those around the right ones (Fig. 2a), whereas the levels of 11β-HSD2 mRNA were slightly, but not significantly, lower in the former than in the latter (Fig. 2b). Therefore, the ratio of 11β-HSD1 versus 11β-HSD2 mRNA was significantly higher in the former than in the latter (Fig. 2c), which is consistent with the results we recently reported using human periodontal tissues.

**Fig. 3:**
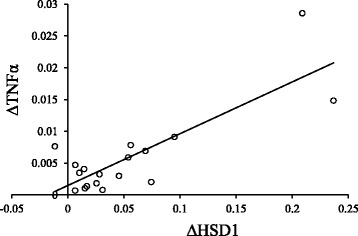
Positive correlation between the increase in the levels of 11β-HSD1 mRNA and that of TNFα mRNA in the ligature-induced periodontitis compared with the control. ΔHSD1: subtraction of the values of 11β-HSD1 mRNA in the control from those in the ligature-induced periodontitis. ΔTNFα: subtraction of the values of TNFα mRNA in the control from those in the ligature-induced periodontitis

The increase in 11β-HSD1 mRNA in the ligature-induced periodontitis compared with the control was positively correlated with that in TNFα mRNA (*p* = 0.000 by significance testing of Pearson’s correlation coefficient) (Fig. 3).

**Fig. 4:**
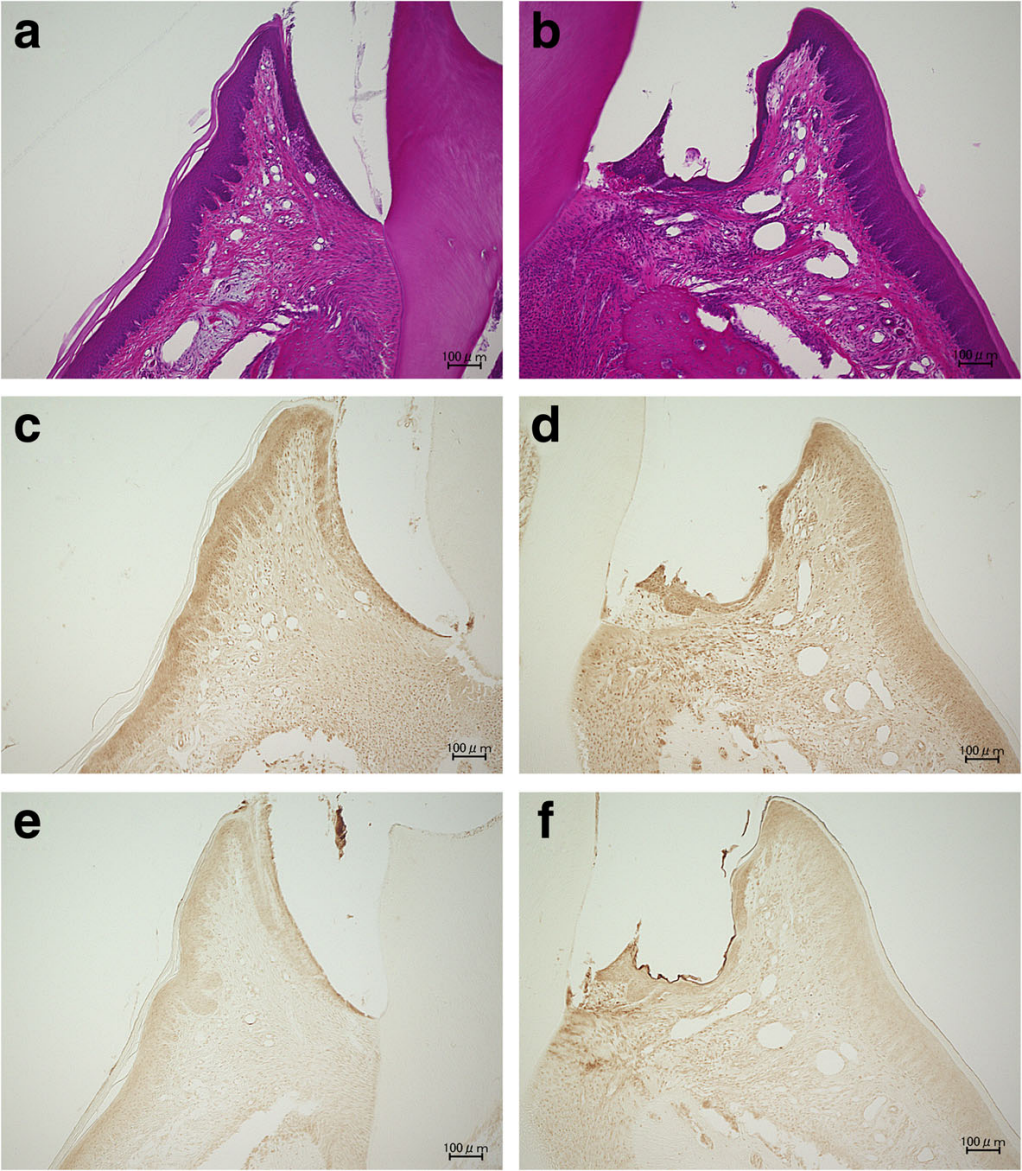
Histological examination of 11β-HSD1 and 11β-HSD2 expression (at lower magnification). **a**, **b** HE staining. **c**, **d** Immunostaining with anti-11β-HSD1 antibody. **e**, **f** Immunostaining with anti-11β-HSD2 antibody. **a**, **c**, **e** Control. **b**, **d**, **f** Ligature-induced periodontitis

**Fig. 5:**
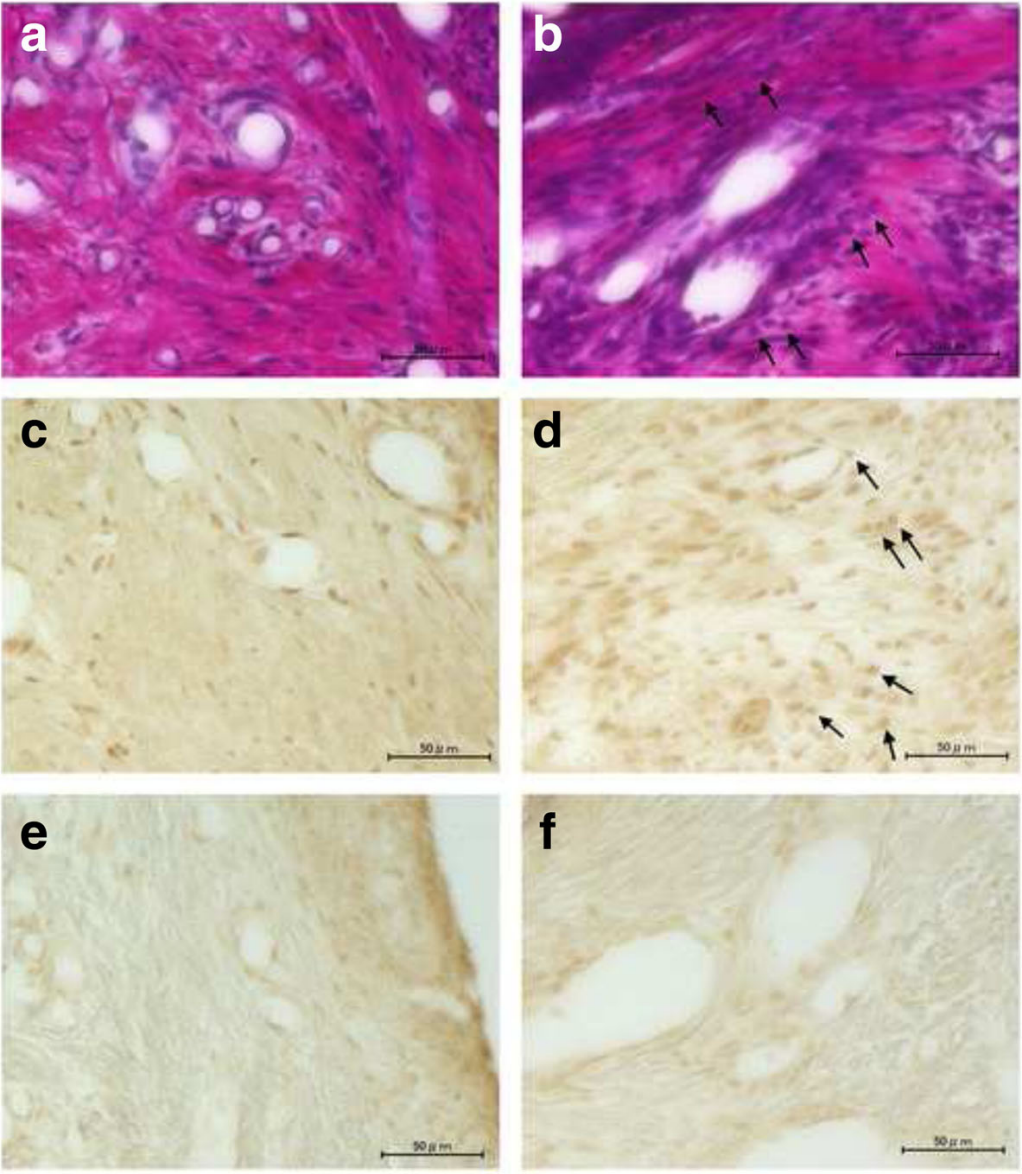
Histological examination of 11β-HSD1 and 11β-HSD2 expression (at higher magnification). **a**, **b** HE staining. **c**, **d** Immunostaining with anti-11β-HSD1 antibody. **e**, **f** Immunostaining with anti-11β-HSD2 antibody. **a**, **c**, **e** Control. **b**, **d**, **f** Ligature-induced periodontitis. Some of the infiltrating neutrophils, which are immunostained with anti-11β-HSD1 antibody, are indicated by the arrows

Histological examinations revealed that the expression of 11β-HSD1 protein was increased in the ligature-induced periodontitis, especially in the infiltrating neutrophils in gingival lamina propria, compared with the control (Figs. 4 and 5), which is in accordance with a recent report demonstrating that 11β-HSD1 is highly expressed in neutrophils [33]. The expression of 11β-HSD2 protein was not apparently different between the control and ligature-induced periodontitis.

## Discussion

The close relationship between increased glucocorticoids and chronic periodontitis has been reported. Since glucocorticoids are increased during stress due to activation of the HPA axis, several studies have reported that chronic periodontitis is associated with stress [1, 2, 5]. In contrast, we have recently reported that human chronic periodontitis is associated with the increased expression of 11β-HSD1, as well as the increased ratio of 11β-HSD1 versus 11β-HSD2 mRNA, which would result in the increased levels of intracellular glucocorticoids [31]. In this study, we demonstrated that the expression of 11β-HSD1, as well as the ratio of 11β-HSD1 versus 11β-HSD2 mRNA, is increased in ligature-induced periodontitis in rats. These results suggest that the increased intracellular glucocorticoids may play a role in the pathogenesis of chronic periodontitis independent of the HPA axis.

11β-HSD1 has been reported to be involved in metabolic diseases, including obesity [13–15]. However, we recently reported that increases in 11β-HSD1 mRNA, as well as the ratio of 11β-HSD1 versus 11β-HSD2 mRNA, were associated with chronic periodontitis, irrespective of obesity in human subjects [31]. Since obesity, as well as metabolic diseases, can be regarded as chronic inflammation of adipose tissue [25, 26], and the increased expression of 11β-HSD1 has been demonstrated in chronic inflammatory diseases such as inflammatory bowel diseases and rheumatoid arthritis [27–30], these results suggest that the increased 11β-HSD1 plays a role in chronic inflammation. The increased expression of 11β-HSD1 in chronic periodontitis in human subjects, as well as in ligature-induced periodontitis in rats, seems to be in accordance with this notion.

The precise role of the increased 11β-HSD1 in chronic inflammation remains to be determined. Although glucocorticoids have strong anti-inflammatory effects [34] pro-inflammatory effects of glucocorticoids have been reported, especially in chronic inflammation [12, 30]. Furthermore, the pro-inflammatory role of 11β-HSD1 have been demonstrated [35, 36]. Thus, overexpression of 11β-HSD1 augmented TNFα-induced iNOS, IL-6, and MCP-1 expression, whereas 11β-HSD1 inhibitors attenuated TNFα-induced NF-kB and MAPK signaling pathways. In this study, the increase in the levels of 11β-HSD1 mRNA was proportional to that of TNFα mRNA, indicating the extent of inflammation (Fig. 3), which is consistent with the pro-inflammatory role of 11β-HSD1 and glucocorticoids. However, the causal relationship between the increased 11β-HSD1 expression and chronic inflammation, including chronic periodontitis, needs to be determined using specific 11β-HSD1 inhibitors or gene knockout animals.

## Conclusions

We demonstrated for the first time that, to the best of our knowledge, the expression of 11β-HSD1 mRNA is increased in experimental periodontitis in animals. This is in agreement with our recent report that the expression of 11β-HSD1 is increased in chronic periodontitis in human subjects irrespective of obesity, suggesting that the increased expression of 11β-HSD1, which would result in in the increased levels of intracellular glucocorticoids, may play a role in the pathogenesis of chronic periodontitis independent of the HPA axis. Further investigation is required to elucidate the precise role of 11β-HSD1 in chronic periodontitis.

## Abbreviations

11β-HSD1: 11β-hydroxysteroid dehydrogenase type 1
11β-HSD2: 11β-hydroxysteroid dehydrogenase type 2
GAPDH: Glyceraldehyde-3-phosphate dehydrogenase
HE: Hematoxylin-eosin
HPA: Hypothalamic-pituitary-adrenal
PCR: Polymerase chain reaction
RT: Reverse transcription
